# Apigenin as a Multitarget Anticancer Agent: Coordinated Inhibition of EGFR/MAPK and PI3K/Akt Signaling in Hematologic Malignancies

**DOI:** 10.3390/ijms27114657

**Published:** 2026-05-22

**Authors:** Hatice Terzi, Şeyma Taştemur, Ayşegül Öztürk, Neslihan Başgöz Karaguş, Mustafa Eymen Kontaş, Onur Mahmutoğlu, Ali Güngör, Mehmet Şencan

**Affiliations:** 1 Department of Internal Medicine, Department of Hematology, Faculty of Medicine, Sivas Cumhuriyet University, 58140 Sivas, Türkiye; mdkontas@gmail.com (M.E.K.); msencan@cumhuriyet.edu.tr (M.Ş.); 2 Department of Internal Medicine, Faculty of Medicine, Sivas Cumhuriyet University, 58140 Sivas, Türkiye; seymatastemur@cumhuriyet.edu.tr; 3 Department of Therapy and Rehabilitation, Vocational School of Health Services, Sivas Cumhuriyet University, 58140 Sivas, Türkiye; aysegulozturk@cumhuriyet.edu.tr; 4 Department of Medical Genetics, Faculty of Medicine, Erciyes University, 38280 Kayseri, Türkiye; neslihanbasgoz@gmail.com; 5 Department of Family Medicine, Sivas Provincial Health Directorate, 58080 Sivas, Türkiye; mdonurmahmutoglu@gmail.com; 6 Laboratory and Veterinary Health Program, Health Services Vocational School, Osmaniye Korkut Ata University, 80000 Osmaniye, Türkiye; aligungor@osmaniye.edu.tr

**Keywords:** apigenin, growth signaling pathways, apoptosis, hematological malignancies, K562, DOHH2

## Abstract

Hematologic malignancies are driven by dysregulated growth and survival signaling pathways that promote proliferation, treatment resistance, and disease progression. Naturally derived compounds targeting multiple oncogenic pathways with low toxicity have gained interest. Apigenin, a dietary flavonoid, shows anticancer activity in solid tumors, but its molecular effects in hematologic malignancies remain unclear. The antineoplastic effects of apigenin were evaluated in K562 (chronic myeloid leukemia) and DOHH2 (B-cell lymphoma) cell lines. Cell viability was assessed using the CCK-8 assay. L929 (mouse fibroblast) cells were included to evaluate selectivity. EGFR, MAPK, PI3K, NF-κB, caspase-3, and caspase-7 levels were measured by ELISA. Apoptosis and cell cycle distribution were analyzed by flow cytometry. Apigenin reduced cell viability in a dose-dependent manner, with IC_50_ values of 84.14 μM (K562) and 70.11 μM (DOHH2). It suppressed EGFR, MAPK, PI3K, and NF-κB signaling and increased the caspase-3 and caspase-7 levels (*p* < 0.001). Flow cytometry showed S-phase arrest and increased apoptosis. L929 cells showed limited reduction in viability at higher concentrations. Apigenin exerts antiproliferative and pro-apoptotic effects via inhibition of the EGFR/MAPK and PI3K/Akt pathways and activation of caspase-mediated apoptosis. Lower sensitivity in L929 cells suggests relative selectivity, supporting further in vivo and clinical studies.

## 1. Introduction

Hematologic malignancies constitute a group of diseases with high mortality rates, arising through different mechanisms at the genetic and molecular levels. Chronic myeloid leukemia (CML) is a clonal myeloproliferative neoplasm characterized by constitutive activation of the BCR: ABL1 fusion oncoprotein. Although most patients are diagnosed during the chronic phase of the disease, progression to the highly aggressive blast phase occurs in approximately 1–2% of cases [1,2]. The advent of first- and subsequent-generation tyrosine kinase inhibitors (TKIs) has significantly reduced CML-related mortality, from approximately 10–20% to around 1–2% per year, bringing survival outcomes closer to those of the general population. Although all TKIs offer comparable survival advantages, their distinct toxicity profiles should guide treatment choice, and strict adherence is crucial to maintain therapeutic efficacy. The greatest challenge for clinicians managing CML is the group of patients who develop intolerance to TKIs, show resistance to treatment, or progress to the blast phase. In these cases, allogeneic hematopoietic stem cell transplantation is currently considered one of the main treatment alternatives [3]. However, it is also known that this approach carries a high risk of treatment-related mortality and morbidity [4]. Therefore, the need to develop novel therapeutic strategies with lower toxicity profiles to prevent both drug resistance and disease progression remains highly relevant in hematologic malignancies including CML. Recent advances in molecularly targeted therapies, immune-based approaches, and pathway-directed treatment strategies have significantly reshaped the therapeutic landscape of hematologic cancers; however, treatment resistance and toxicity continue to represent major clinical challenges [2,5].

**Figure 1:**
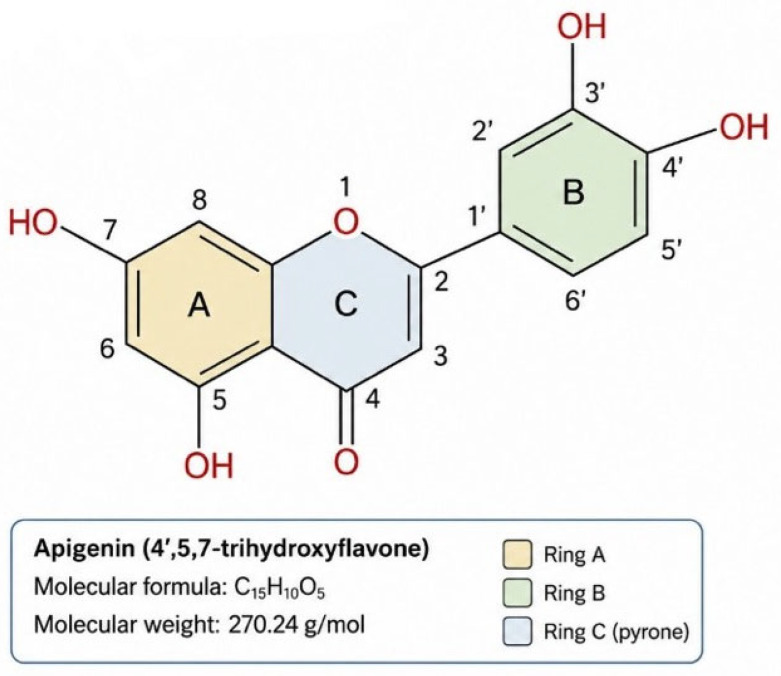
Chemical structure of apigenin (4′,5,7-trihydroxyflavone).

Lymphomas, which occupy an important place among hematological malignancies, constitute a highly heterogeneous group with respect to their clinical, morphological, and molecular characteristics. Diffuse large B-cell lymphoma (DLBCL) constitutes the most common and biologically aggressive form of non-Hodgkin lymphoma among adults [6]. Although the standard R-CHOP chemotherapy protocol is effective in the majority of patients, relapse or refractory disease may occur in certain patient groups [7]. Although it is a subtype known to be sensitive to chemotherapy, the management of relapse/refractory cases and the clinical follow-up of the fragile patient group due to high treatment toxicity pose a significant challenge for clinicians. Taken together, these limitations highlight the need for novel therapeutic approaches with improved efficacy and lower treatment-related toxicity. Recent advances in molecularly targeted therapies, immune-based approaches, and pathway-directed treatment strategies have substantially reshaped the therapeutic landscape of hematologic malignancies. Nevertheless, treatment resistance, disease progression, and therapy-related toxicity continue to represent major clinical challenges, particularly in aggressive and refractory hematologic cancers [5,8,9]. Consequently, the identification and preclinical assessment of innovative, mechanism-oriented therapeutic approaches for the treatment of DLBCL have become a central priority in modern hematologic oncology research. In parallel, naturally derived bioactive compounds have attracted growing scientific interest due to their comparatively low toxicity and their ability to regulate multiple oncogenic signaling pathways concurrently. Among these compounds, apigenin (4′,5,7-trihydroxyflavone) is a particularly noteworthy example (Figure 1).

**Figure 2:**
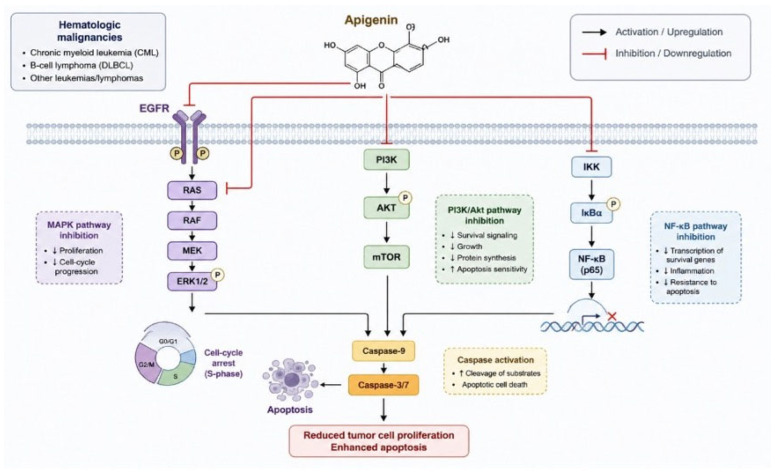
Proposed molecular mechanisms of apigenin in hematologic malignancies. Apigenin suppresses the EGFR/MAPK and PI3K/Akt signaling pathways, inhibits NF-κB activity, induces S-phase cell-cycle arrest, and activates caspase-mediated apoptosis, resulting in reduced tumor cell proliferation and enhanced apoptotic cell death.

To provide a mechanistic overview of the molecular pathways investigated in the present study, a schematic illustration summarizing the proposed effects of apigenin on EGFR/MAPK and PI3K/Akt signaling cascades has been included (Figure 2). EGFR-mediated signaling plays a central role in regulating tumor cell proliferation, survival, cell-cycle progression, and resistance to apoptosis through the downstream activation of the MAPK and PI3K/Akt pathways. Dysregulation of these signaling networks contributes to uncontrolled cellular growth and therapeutic resistance in multiple hematologic malignancies [10,11]. Previous experimental evidence suggests that apigenin can suppress EGFR activation, inhibit MAPK and PI3K/Akt signaling, reduce NF-κB activity, and induce caspase-mediated apoptosis [12]. Therefore, simultaneous targeting of these interconnected pathways may represent an important mechanism underlying the antiproliferative and proapoptotic effects of apigenin in leukemia and lymphoma cells. This dietary flavonoid, abundantly found in fruits, vegetables, and medicinal herbs such as parsley, chamomile, and celery, exhibits remarkable anticancer properties mediated through diverse molecular and cellular mechanisms [13]. This molecule exhibits potent anti-inflammatory and antioxidant activities while demonstrating minimal inherent toxicity, characteristics that underscore its potential applicability as an anticancer agent [14,15]. Accumulating evidence indicates that apigenin exhibits broad-spectrum antitumor activity across multiple human cancer models, including breast cancer, colorectal carcinoma, hepatocellular carcinoma, lung cancer, leukemia, and lymphoma [10,12,16]. Previous studies have demonstrated that apigenin suppresses proliferation and induces apoptosis in breast cancer cells with reported IC_50_ values ranging between approximately 10–40 μM, while IC_50_ values of approximately 20–80 μM have been reported in colorectal and hepatocellular carcinoma models depending on exposure duration and cellular context. In hematologic malignancies, apigenin has been shown to induce apoptosis and cell-cycle arrest in leukemia and lymphoma cell lines through modulation of the PI3K/Akt, MAPK, and NF-κB signaling pathways. For example, previous studies demonstrated that apigenin suppresses survival signaling and promotes caspase-dependent apoptosis in diffuse large B-cell lymphoma and leukemia models, supporting its potential role as a multitarget anticancer compound in hematologic cancers [11,17,18].

Numerous studies have further demonstrated that apigenin mediates its anticancer effects across diverse solid tumors by modulating multiple molecular pathways involved in cell-cycle regulation, apoptotic signaling, and metastasis suppression [19,20]. Apigenin reduces cancer cell glucose uptake, inhibits extracellular matrix remodeling, and hinders the development of tumor blood vessels [15]. EGFR and MAPK signaling pathways are key regulators of cell proliferation and the G1/S transition, and their dysregulation promotes uncontrolled DNA synthesis and tumor growth. Apigenin suppresses EGFR phosphorylation, modulates MAPK activity, and downregulates cyclins and CDKs while upregulating p21 and p27, leading to cell cycle arrest and apoptosis. Studies have shown that apigenin inhibits G1/S transition and DNA replication in various cancer models, highlighting its therapeutic potential against EGFR/MAPK-driven malignancies [11,19]. Its differential effects on normal versus cancerous cells and potential as an adjuvant chemotherapeutic agent make apigenin a promising candidate for cancer prevention and treatment [13,21]. Therefore, evaluation of its effects on normal cells is essential to better define its therapeutic selectivity.

In the present investigation, the impact of apigenin on cellular viability, apoptotic induction, cell-cycle regulation, and proliferative signaling cascades was systematically assessed in the K562 and DOHH2 cell lines to delineate its therapeutic potential in hematologic malignancies, with additional evaluation in a normal fibroblast cell line to assess relative selectivity.

## 2. Results

### 2.1. Effects of Apigenin on Cell Viability of L929, K562, and DOHH2 Cells

**Figure 3:**
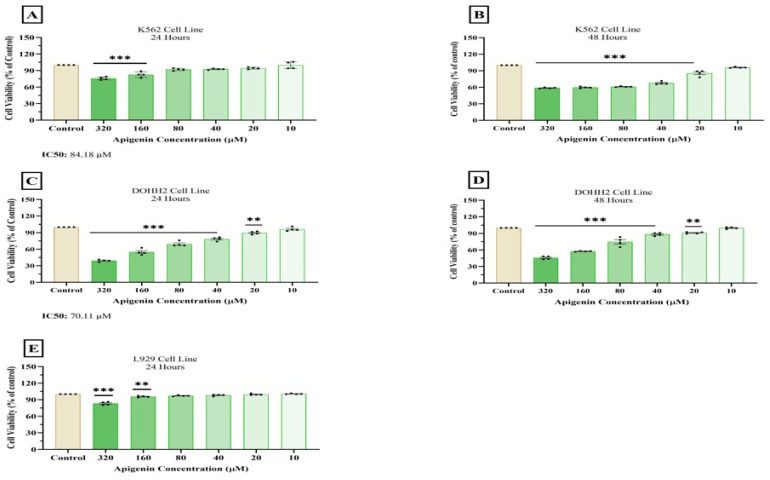
Effects of apigenin treatment on cell viability in (**A**,**B**) K562 and (**C**,**D**) DOHH2 cell lines at 24 and 48 h. Effects of apigenin on cell viability in L929 normal fibroblast cells at 24 and 48 h (**E**). Data are presented as mean ± SEM. ** *p* < 0.01 and *** *p* < 0.001 compared to the control group (n = 4). In addition to its cytotoxic effects, apigenin induced notable morphological changes in both cell lines, including cell shrinkage, increased rounding, and reduced cellular density relative to the control cultures, morphological features consistent with apoptotic cell death (Figure 4A,B). In contrast, L929 cells exhibited only a modest decrease in viability, primarily at higher concentrations, with minimal effects observed at lower doses, indicating relative selectivity toward malignant cells.

The effect of apigenin on the viability of the L929, K562, and DOHH2 cell lines was assessed using the CCK-8 assay at concentrations of 10, 20, 40, 80, 160, and 320 μM over 24 and 48 h. Apigenin treatment caused a significant, concentration- and time-dependent reduction in cell viability in both cell lines compared to the untreated controls (*p* < 0.001; Figure 3). After 24 h of exposure, the calculated half-maximal inhibitory concentration (IC_50_) values were 84.14 μM for K562 cells (Figure 3A) and 70.11 μM for DOHH2 cells (Figure 3C). The inhibitory effect of apigenin was sustained and further enhanced after 48 h of treatment in both K562 (Figure 3B) and DOHH2 (Figure 3D) cells, particularly at higher concentrations. In contrast, apigenin induced only a limited reduction in cell viability in the L929 normal fibroblast cell line, with noticeable effects primarily observed at the highest concentrations and minimal changes at lower doses (Figure 3E). This differential response suggests that apigenin exerts relatively selective cytotoxic effects on malignant hematologic cell lines compared to normal cells. Overall, these findings indicate that apigenin moderately suppresses the proliferation of K562 and DOHH2 cells in a dose- and time-dependent manner.

**Figure 4:**
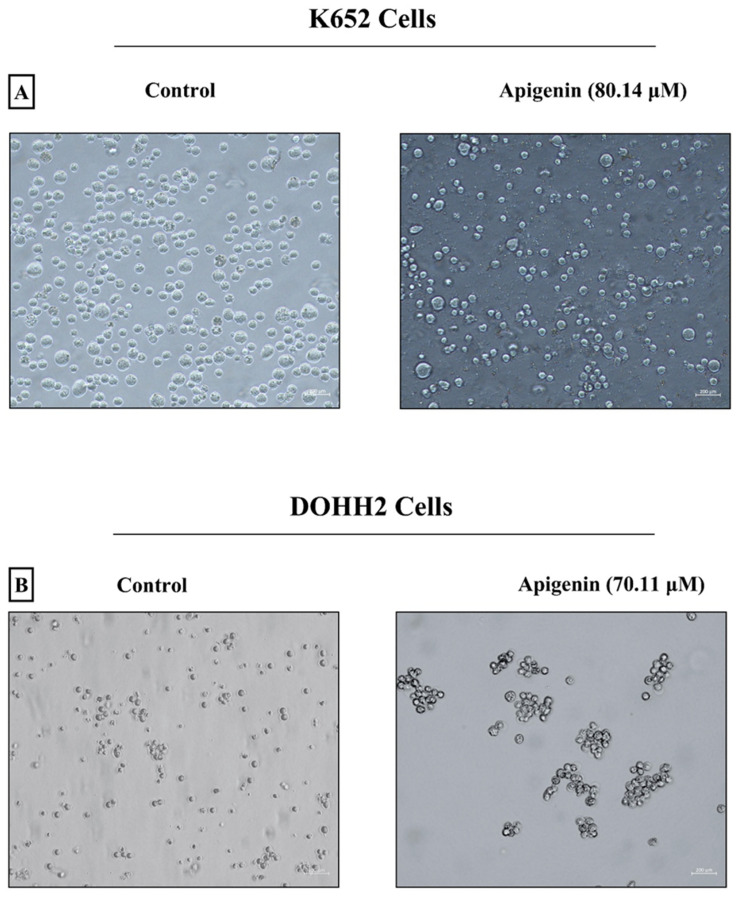
Effects of apigenin treatment on cell morphology in (**A**) K562 and (**B**) DOHH2 cells. Apigenin was applied at concentrations of 84.14 μM for K562 cells and 70.11 μM for DOHH2 cells for 24 h. Morphological changes were examined microscopically using an inverted microscope at 20× magnification and compared with the control group.

### 2.2. Effects of Apigenin on EGFR, MAPK, PI3K, NF-κB, Caspase-7 and Caspase-3 in K562 and DOHH2 Cells

**Figure 5:**
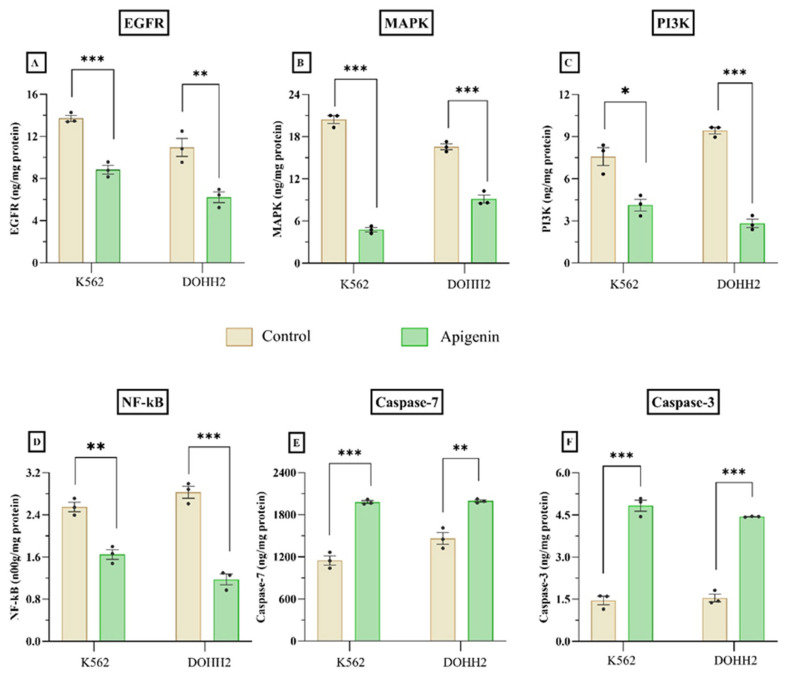
Effects of apigenin treatment on (**A**) EGFR, (**B**) MAPK, (**C**) PI3K, (**D**) NF-κB, (**E**) caspase-7, and (**F**) caspase-3 levels in K562 and DOHH2 cells. Protein levels were determined using ELISA kits. Data are presented as the mean ± SEM. * *p* < 0.05, ** *p* < 0.01, and *** *p* < 0.001 compared to the control group (n = 3).

Apigenin treatment significantly suppressed signaling pathways associated with proliferation and survival in both cell lines, while increasing the markers of apoptosis (*p* < 0.001; Figure 5).

K562 cells exhibited significantly reduced levels of EGFR (*p* < 0.001; Figure 5A), MAPK (*p* < 0.001; Figure 5B), PI3K (*p* < 0.05; Figure 5C), and NF-κB (*p* < 0.01; Figure 5D) compared to the control group. Concurrently, significant increases were observed in caspase-7 (*p* < 0.001; Figure 5E) and caspase-3 (*p* < 0.001; Figure 5F), both of which are markers of apoptosis.

Similarly, in the DOHH2 cells, significant decreases were observed in the levels of EGFR (*p* < 0.01; Figure 5A), MAPK (*p* < 0.001; Figure 5B), PI3K (*p* < 0.001; Figure 5C), and NF-κB (*p* < 0.01; Figure 5D). Conversely, the levels of caspase-7 (*p* < 0.01; Figure 5E) and caspase-3 (*p* < 0.001; Figure 5F), which indicate an apoptotic response, were significantly elevated. These findings suggest that apigenin promotes apoptotic responses while suppressing proliferation-related signaling pathways.

### 2.3. Effects of Apigenin on Cell Cycle Distribution in K562 and DOHH2 Cells

**Figure 6:**
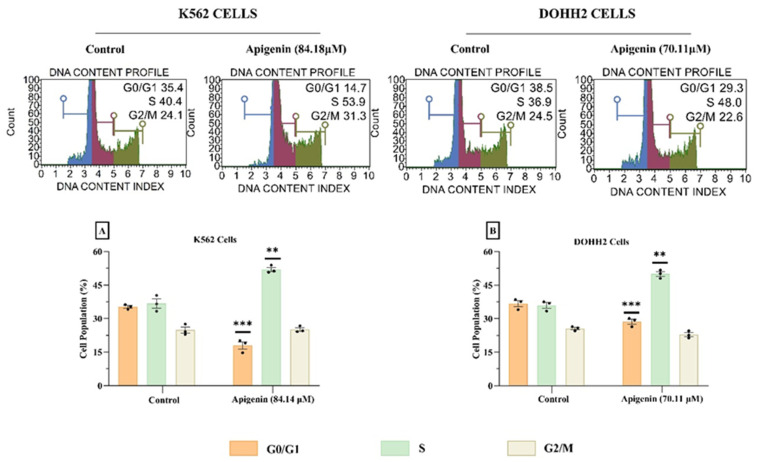
Effects of apigenin treatment on cell cycle progression in (**A**) K562 and (**B**) DOHH2 cells. Quantitative analysis was performed using flow cytometry, and data are expressed as the mean ± SEM. ** *p* < 0.01, and *** *p* < 0.001 compared to the control group (n = 3). (Blue: GO/G1, Pink: S, Green: G2/M).

Cell-cycle profiling of K562 and DOHH2 cells demonstrated that exposure to apigenin led to a substantial accumulation of cells in the S phase. In both cell models, the proportion of S-phase cells was significantly increased relative to the untreated controls (*p* < 0.001; Figure 6A,B). These data indicate that apigenin exerts an antiproliferative effect, at least in part, through the induction of S-phase cell-cycle arrest.

### 2.4. Effect of Apigenin on Apoptosis in K562 and DOHH2 Cells Assessed by Annexin V Staining

The proapoptotic effects of apigenin were quantitatively evaluated in K562 and DOHH2 cells using Annexin V flow cytometry. In K562 cells, treatment with 84.14 μM apigenin for 24 h significantly decreased the proportion of live cells from 90.26 ± 0.49% to 54.19 ± 0.73% (*p* < 0.001). Early apoptosis increased from 2.21 ± 0.15% to 19.00 ± 0.27%, and late apoptosis increased from 5.07 ± 0.16% to 25.51 ± 0.45% (*p* < 0.001). The percentage of dead cells did not change significantly (*p* > 0.05).

Similarly, in DOHH2 cells, 24-h treatment with 70.11 μM apigenin reduced the proportion of live cells from 88.39 ± 0.43% to 56.68 ± 1.11%, while early and late apoptotic cells increased to 21.15 ± 0.63% and 20.01 ± 1.15%, respectively.

**Figure 7:**
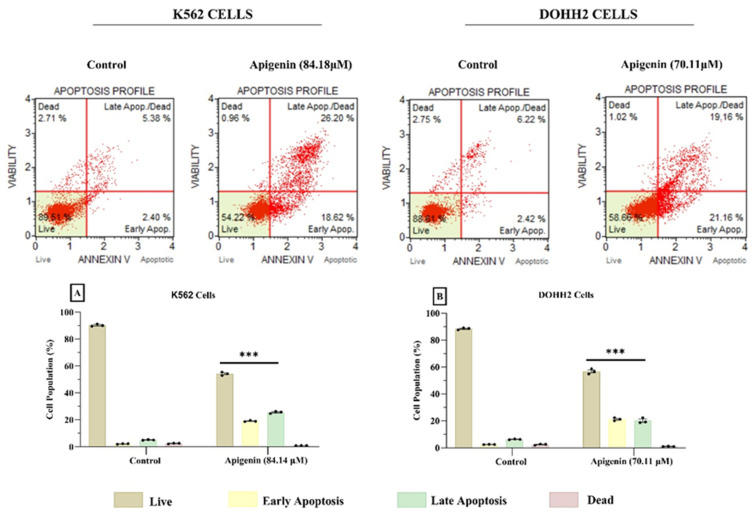
The administration of apigenin affects apoptosis, as measured by the Annexin V binding assay, in (**A**) K562 and (**B**) DOHH2 cells. Flow cytometry histograms illustrate the apoptosis rates in (**A**) K562 and (**B**) DOHH2 cells, accompanied by a graphical presentation of apoptosis percentages. Data are presented as mean ± SEM. *** *p* < 0.001 compared to the control group (n = 3).

These results indicate that apigenin primarily induces apoptosis rather than necrosis in both cell lines. Representative Annexin V flow cytometry histograms and quantitative apoptosis analyses are presented in Figure 7.

### 2.5. Effect of Apigenin on EGFR and Cleaved Caspase-3 Expression in K562 and DOHH2 Cells Assessed by Immunofluorescence

**Figure 8:**
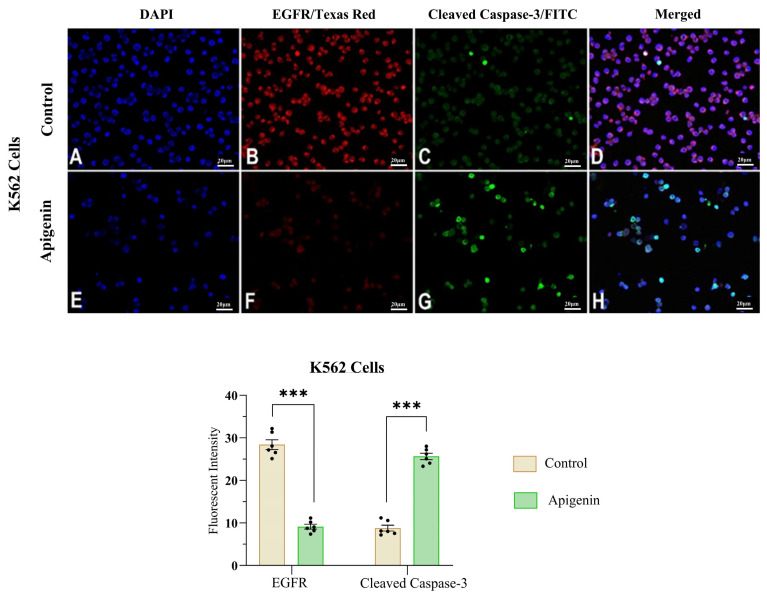
Effects of apigenin on EGFR and cleaved caspase-3 expression in K562 cells. Representative immunofluorescence images of (**A**,**E**) DAPI (blue), (**B**,**F**) EGFR (Texas Red), (**C**,**G**) cleaved caspase-3 (FITC), and (**D**,**H**) merged images in the control and apigenin-treated groups. Apigenin significantly decreased EGFR and increased cleaved caspase-3 expression. Data are presented as mean ± SEM (six random fields per group). *** *p* < 0.001 vs. control. Scale bar: 20 μm.

**Figure 9:**
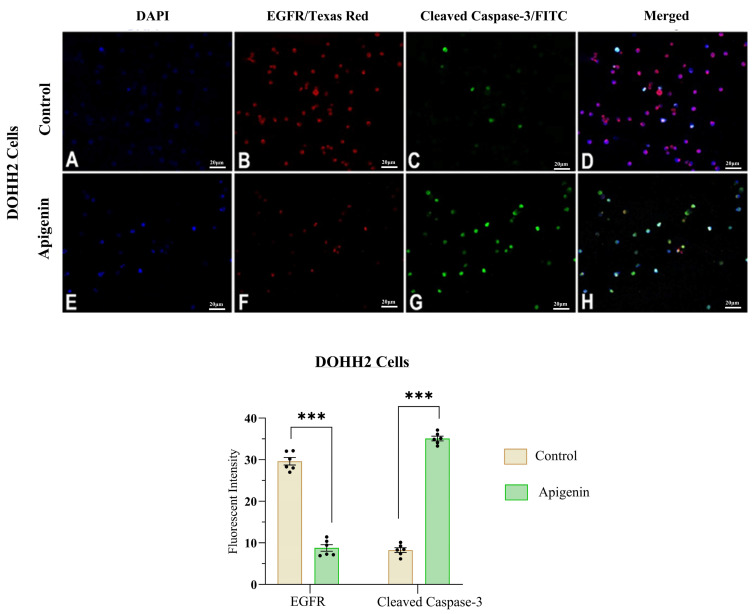
Effects of apigenin on EGFR and cleaved caspase-3 expression in DOHH2 cells. Representative immunofluorescence images showing the expression of (**A**,**E**) DAPI (blue), (**B**,**F**) EGFR (Texas Red), (**C**,**G**) cleaved caspase-3 (FITC), and (**D**,**H**) merged images in the control and apigenin-treated groups. Quantitative analysis of fluorescence intensity demonstrated a significant downregulation of EGFR and a marked upregulation of cleaved caspase-3 following apigenin treatment. Data are presented as the mean ± SEM from six random fields per group. *** *p* < 0.001 vs. control. Scale bar: 20 μm.

To elucidate the molecular mechanisms underlying apigenin-induced growth inhibition, the expression levels of EGFR and cleaved caspase-3 were analyzed in K562 and DOHH2 cells using immunofluorescence staining (Figure 8 and Figure 9). In control K562 cells, strong EGFR immunoreactivity (red) was predominantly observed on the cell membrane and in the cytoplasm, whereas cleaved caspase-3 expression (green) was minimal. Apigenin treatment significantly reduced the EGFR fluorescence intensity (*p* < 0.001; Figure 8B,F) while markedly increasing cleaved caspase-3 immunopositivity (*p* < 0.001; Figure 8C,G). These results suggest that apigenin exerts its antiproliferative effects by suppressing EGFR signaling and inducing caspase-mediated apoptosis in K562 cells.

Immunofluorescence staining was performed on DOHH2 cells (Figure 9). Similar to the K562 cells, the control DOHH2 cells exhibited high EGFR expression (red) and minimal cleaved caspase-3 immunoreactivity (green). Apigenin treatment significantly reduced the EGFR fluorescence intensity (*p* < 0.001; Figure 9B,F) while markedly increasing cleaved caspase-3 expression (*p* < 0.001; Figure 9C,G). These findings indicate that apigenin suppresses EGFR signaling and induces caspase-mediated apoptosis in DOHH2 cells, supporting its potential as a targeted therapeutic agent.

## 3. Discussion

Apigenin is a naturally occurring flavonoid widely distributed in fruits, vegetables, and medicinal plants, and it has attracted considerable attention for its broad spectrum of anticancer properties. Several experimental studies have demonstrated that apigenin exerts antiproliferative and proapoptotic effects in both solid and hematologic malignancies by modulating multiple oncogenic signaling pathways, including PI3K/Akt, MAPK, and NF-κB. These multitarget effects have been demonstrated in various malignancies, including hematologic cancers [14,20,22,23].

In the present study, we investigated the antitumor effects of apigenin in two hematologic malignancy cell lines, K562 (chronic myeloid leukemia) and DOHH2 (B-cell lymphoma). Our results demonstrated that apigenin induced a measurable reduction in cell viability in both hematologic malignancy cell lines in a dose-dependent manner. Notably, the calculated IC_50_ values indicated that DOHH2 cells were more sensitive to apigenin treatment than K562 cells (70.11 μM vs. 84.14 μM). Therefore, the higher sensitivity observed in DOHH2 cells primarily reflects lower IC_50_ values and a more pronounced apoptotic response in this lymphoma cell line. Previous experimental studies investigating hematologic malignancies support the molecular findings observed in the present study. Ruela-de-Sousa et al. demonstrated that apigenin induces apoptosis and suppresses proliferation in leukemia cell models through the activation of caspase-dependent pathways and modulation of survival signaling [16]. Similarly, Huang et al. reported that apigenin significantly inhibited proliferation and promoted apoptosis in diffuse large B-cell lymphoma cells by suppressing the PI3K/Akt and NF-κB signaling pathways [10]. Additional mechanistic studies further demonstrated that apigenin-mediated inhibition of MAPK and Akt signaling contributes to impaired tumor cell proliferation, increased apoptotic susceptibility, and disruption of cell-cycle progression in hematologic cancer models [11]. These previously reported findings are highly consistent with the molecular alterations observed in the present study.

Importantly, this antiproliferative effect was further enhanced at 48 h, indicating a time-dependent intensification of apigenin-mediated cytotoxicity. As demonstrated in the viability assays, prolonged exposure resulted in a more pronounced reduction in cell viability in both K562 and DOHH2 cells, particularly at higher concentrations. This observation is consistent with previous studies reporting that apigenin exerts cumulative anticancer effects through the sustained suppression of survival signaling pathways and progressive activation of apoptotic cascades [19,23,24]. The time-dependent increase in cytotoxicity suggests that apigenin induces not only transient growth inhibition but also durable impairment of cellular proliferation capacity through continuous pathway inhibition. Consistent with the viability data, Annexin V analysis revealed a significant increase in early and late apoptotic cell populations following apigenin treatment in both cell lines (Figure 7). Apoptosis induction represents one of the most well-established anticancer mechanisms of apigenin. Previous in vitro studies have demonstrated that apigenin promotes apoptosis by activating caspase-dependent pathways and modulating mitochondrial apoptotic signaling [20,25]. For instance, Shukla and Gupta reported that apigenin induces apoptosis by activating caspase cascades and disrupting mitochondrial membrane potential in cancer cells [22]. Similarly, Patel et al. demonstrated that apigenin can trigger apoptosis by regulating Bcl-2 family proteins and activating caspase-3-dependent pathways [26]. In agreement with these findings, our results showed increased levels of caspase-3 and caspase-7 following apigenin treatment, supporting the involvement of caspase-mediated apoptosis in the cytotoxic effects observed in both leukemia and lymphoma cell models.

Another important mechanism underlying the anticancer activity of apigenin is its ability to regulate cell-cycle progression [27,28]. In the present study, cell-cycle analysis demonstrated that apigenin treatment resulted in a significant accumulation of cells in the S phase in both K562 and DOHH2 cell lines, indicating interference with DNA replication and cellular proliferation. Similar observations have been reported in several previous experimental studies. For example, apigenin has been shown to induce cell-cycle arrest through the regulation of cyclins, cyclin-dependent kinases, and checkpoint proteins, thereby inhibiting tumor cell proliferation [14,19]. Depending on the cellular context, apigenin has been reported to induce G2/M or S-phase arrest, suggesting that disruption of cell-cycle regulation represents an important component of its antiproliferative activity [28,29,30,31]. These findings indicate that apigenin exerts its antitumor effects not only through apoptosis induction but also by interfering with cell-cycle control mechanisms.

At the molecular level, apigenin has been shown to modulate multiple oncogenic signaling pathways involved in tumor cell survival and proliferation. Among these pathways, the PI3K/Akt signaling pathway plays a central role in regulating cell survival, metabolism, and resistance to apoptosis. Previous studies have demonstrated that apigenin suppresses PI3K/Akt signaling, thereby promoting apoptotic cell death and inhibiting tumor cell proliferation [19,23]. In addition, apigenin has been reported to inhibit the MAPK signaling pathway, particularly ERK1/2 activation, which plays an important role in regulating cell proliferation and mitogenic signaling [18,32]. Furthermore, apigenin can suppress NF-κB signaling, a transcription factor that regulates genes involved in inflammation, tumor progression, and resistance to apoptosis [24,32]. The ability of apigenin to simultaneously modulate these signaling pathways highlights its potential role as a multi-target anticancer compound [19,24].

Consistent with these previously reported mechanisms, our findings demonstrated that apigenin significantly reduced the expression of EGFR, MAPK, PI3K, and NF-κB signaling components in both K562 and DOHH2 cells. Although phosphorylation-specific analyses were not performed in the present study, the observed downregulation of total EGFR, MAPK, and PI3K protein levels strongly suggests a functional suppression of their downstream signaling cascades. Previous studies have consistently demonstrated that apigenin inhibits the activation of key signaling mediators, including phosphorylated AKT (p-AKT) and phosphorylated ERK (p-ERK), which represent critical nodes within the PI3K/Akt and MAPK pathways, respectively. In particular, apigenin-mediated suppression of p-AKT has been associated with reduced cell survival and increased apoptotic susceptibility, while the inhibition of p-ERK signaling has been linked to impaired proliferative capacity and cell-cycle arrest. Therefore, although phosphorylation status was not directly evaluated in this study, the present findings are in strong agreement with the existing literature and support the notion that apigenin exerts its anticancer effects through inhibition of these key oncogenic signaling axes. Consistent with these previously reported mechanisms, our findings demonstrated that apigenin reduced EGFR, MAPK, PI3K, and NF-κB signaling components in both K562 and DOHH2 cells. Similar observations have been reported in previous experimental studies. Previous investigations demonstrated that apigenin suppresses PI3K/Akt and NF-κB signaling, induces caspase-dependent apoptosis, and inhibits proliferation in leukemia and diffuse large B-cell lymphoma models [10,16]. These findings collectively support the concept that coordinated suppression of survival signaling pathways represents a central mechanism underlying the anticancer effects of apigenin in hematologic malignancies. The coordinated suppression of these signaling pathways, together with increased caspase activation and disruption of cell-cycle progression, suggests that apigenin exerts its anticancer effects through a complex network of molecular mechanisms.

In this context, the immunofluorescence findings presented in Figure 8 and Figure 9 provide important spatial confirmation of these molecular alterations. Apigenin treatment resulted in a marked reduction in EGFR fluorescence intensity, accompanied by a significant increase in cleaved caspase-3 expression in both K562 and DOHH2 cells. These results are consistent with the biochemical analyses and demonstrate that pathway modulation occurs not only at the protein level but also at the cellular and morphological levels. Given that EGFR is a key upstream regulator of MAPK and PI3K/Akt signaling, its downregulation is strongly associated with the suppression of proliferative signaling and increased susceptibility to apoptosis [18,19].

Moreover, the increased cleaved caspase-3 immunoreactivity confirms activation of the execution phase of apoptosis, a critical step in programmed cell death [29]. The simultaneous downregulation of EGFR and activation of caspase-3 suggest that apigenin effectively shifts the cellular balance from survival signaling toward apoptotic pathways. Similar coordinated effects have been reported in previous studies, supporting the role of apigenin as a potent modulator of growth factor signaling and apoptosis in cancer cells [18,25]. Importantly, the slightly greater sensitivity observed in DOHH2 lymphoma cells compared with K562 leukemia cells may reflect differences in pathway dependency between these malignancies. B-cell lymphomas frequently rely on PI3K and NF-κB signaling pathways for survival and proliferation. Therefore, stronger inhibition of these pathways by apigenin may partly explain the greater responsiveness observed in lymphoma cells in our study. However, further mechanistic studies are required to clarify the molecular basis of this differential sensitivity.

From a therapeutic and translational perspective, these findings should be interpreted with caution. Although tyrosine kinase inhibitors have significantly improved outcomes in chronic myeloid leukemia, therapeutic resistance and disease progression remain major challenges in several hematologic malignancies, including aggressive lymphomas [33]. In this context, natural compounds such as apigenin have attracted increasing attention due to their ability to simultaneously modulate multiple oncogenic signaling pathways while generally exhibiting low toxicity in experimental systems [14,19,34]. Importantly, the relatively limited reduction in viability observed in L929 normal fibroblast cells under identical experimental conditions suggests a degree of selective cytotoxicity toward malignant hematologic cells. Significantly, the relatively high micromolar IC_50_ values observed in the present study suggest that apigenin exhibits moderate antiproliferative activity in hematologic malignancy models under the tested experimental conditions. In this context, the findings may be particularly valuable in defining the relative sensitivity and selectivity profile of apigenin across different cancer types rather than supporting its use as a highly potent standalone cytotoxic agent. Previous studies conducted in several solid tumor models have reported lower IC_50_ ranges, suggesting that the responsiveness to apigenin may vary substantially depending on tumor biology and pathway dependency [22].

However, given that the present findings are derived exclusively from in vitro models, apigenin may be more appropriately considered as a potential adjunct therapeutic agent rather than a standalone cytotoxic compound. Its capacity to coordinately inhibit key signaling axes, including EGFR/MAPK and PI3K/Akt, indicates that it may enhance the efficacy of existing treatment strategies and help overcome resistance mechanisms. In particular, the multitargeted nature of apigenin may provide synergistic benefits when combined with conventional chemotherapeutic agents or targeted therapies, potentially allowing dose reduction and minimizing treatment-related toxicity. Therefore, further validation in in vivo models and rational combination-based therapeutic studies is required to fully elucidate its clinical applicability. An additional limitation of the present study is that cell viability analyses were restricted to 24- and 48-h exposure periods. Although these time points were sufficient to demonstrate dose- and time-dependent antiproliferative effects, longer exposure durations such as 72 h may provide a more comprehensive evaluation of the sustained cytotoxic and cytostatic effects of apigenin. Future studies incorporating extended treatment periods may further clarify the long-term biological responses and temporal dynamics associated with apigenin exposure in hematologic malignancies.

Several limitations of this study should be acknowledged while also considering the scope and design of the present work. First, the experiments were conducted exclusively in vitro using well-established hematologic cancer cell lines, which represent widely accepted and reproducible preclinical models for mechanistic investigations. Although such systems cannot fully recapitulate the complexity of in vivo tumor biology, they provide a controlled environment to delineate cellular and molecular responses to therapeutic agents.

Second, although significant alterations in multiple oncogenic signaling pathways were demonstrated, phosphorylation-specific analyses and Western blot validation could not be performed due to technical constraints encountered during the experimental phase. We acknowledge that ELISA-based quantification may be associated with methodological limitations and potential assay-related artifacts when evaluating intracellular signaling proteins. To strengthen the reliability of the biochemical findings, immunofluorescence analyses for EGFR and cleaved caspase-3 were additionally performed and demonstrated results consistent with the ELISA data. Nevertheless, future studies incorporating Western immunoblotting and phosphorylation-specific analyses of key signaling mediators, including p-AKT and p-ERK, will be important to further validate the molecular mechanisms proposed in the present study. Importantly, the observed findings are also consistent with previously published studies reporting that apigenin suppresses PI3K/Akt and MAPK pathway activity and induces caspase-mediated apoptosis in hematologic malignancy models.

Another consideration is the relatively high concentrations of apigenin required to achieve cytotoxic effects in vitro. While the IC_50_ values fall within the micromolar range, this is a commonly reported phenomenon for naturally derived compounds, largely reflecting differences in bioavailability, metabolism, and cellular uptake under in vitro conditions. Importantly, the concentration range used in the present study is consistent with several previously published in vitro investigations evaluating apigenin and related flavonoids in cancer models. Previous studies have frequently employed micromolar concentrations ranging between approximately 20–200 μM to evaluate the antiproliferative and proapoptotic effects of apigenin under cell culture conditions. The inclusion of higher concentrations in the present study was intended to comprehensively characterize the dose–response relationship and accurately determine the IC_50_ values in hematologic malignancy cell lines. Furthermore, naturally derived flavonoids such as apigenin are known to exhibit limited aqueous solubility, restricted intracellular accumulation, and relatively low bioavailability in vitro, factors that may necessitate the use of higher experimental concentrations to achieve measurable biological effects. Importantly, such concentrations should not be directly extrapolated to in vivo settings, where pharmacokinetic optimization, targeted delivery systems, and combination strategies may substantially enhance therapeutic efficacy.

Finally, the use of the L929 cell line as a model for normal cells represents a methodological limitation, as these cells are of murine origin and do not fully mimic normal human hematopoietic counterparts. However, L929 fibroblasts are widely utilized as a standard reference model in cytotoxicity studies and provide a reproducible baseline for comparative analyses. The relatively limited cytotoxic effect observed in these cells supports a preliminary indication of selective activity toward malignant cell populations. Future studies incorporating primary human hematopoietic cells and in vivo models will be essential to further validate these findings and strengthen their translational relevance.

## 4. Materials and Methods

### 4.1. Cell Culture and Materials

The K562 cell line (chronic myeloid leukemia blast crisis; CCL-243) was obtained from the American Type Culture Collection (ATCC), whereas the DOHH2 cell line (B-cell lymphoma; ACC-47) was sourced from DSMZ CellDive. The L929 cell line (mouse-derived normal fibroblast cell line; CCL-1) was also obtained from ATCC. Both cell models were cultured in RPMI-1640 medium (Nutriculture, Ecotech Biotechnology, Erzurum, Türkiye) supplemented with 10% fetal bovine serum and 1% penicillin–streptomycin (Sigma-Aldrich, Darmstadt, Germany). Cultures were maintained at 37 °C in a humidified incubator with 5% CO_2_ under standard conditions.

### 4.2. Apigenin Stock Solution and Application Method

Apigenin (BLDpharm, Shanghai, China), a flavonoid characterized by limited aqueous solubility, was dissolved in dimethyl sulfoxide (DMSO) to generate a concentrated stock solution. A 100 mM stock was prepared by dissolving the compound in DMSO and subsequently stored at −20 °C in light-protected containers. Working concentrations for in vitro experiments were freshly obtained by diluting the stock solution in RPMI-1640 culture medium, with the final DMSO concentration carefully maintained below 0.1% to avoid solvent-related cytotoxic effects. The selected concentration range was based on previously published in vitro studies investigating apigenin and structurally related flavonoids in cancer cell models [11,16].

### 4.3. CCK-8 Cell Viability Assay

Cell viability was assessed using the Cell Counting Kit-8 (CCK-8; Abbkine, GA, USA). Cells were seeded in 96-well plates at a density of 5 × 10^3^ cells per well and incubated with apigenin for 24 and 48 h. The L929 normal fibroblast cell line was included and treated under identical experimental conditions to evaluate relative cytotoxic selectivity. Following the treatment period, 10 µL of CCK-8 solution was added to each well and incubated for 4 h at 37 °C to allow sufficient WST-8 reduction within the recommended linear detection range (1–4 h). Absorbance was measured at 450 nm using a microplate reader (Thermo Fisher Scientific, Altrincham, UK), and relative cell viability was calculated accordingly.

### 4.4. Formation of Cell Lysates

For ELISA analyses, both untreated control cells and apigenin-treated cells were included. Cells were exposed to apigenin for 24 h at concentrations corresponding to their respective IC_50_ values determined by the CCK-8 assay (84.14 µM for K562 and 70.11 µM for DOHH2 cells). The IC_50_ concentrations were selected to ensure biologically relevant exposure while maintaining sufficient viable cells for biochemical analyses. Following treatment, cells were collected, transferred to sterile 1.5 mL microcentrifuge tubes, and centrifuged at 2000 rpm for 10 min to obtain cell pellets. After careful removal of the supernatant, the pellets were resuspended in phosphate-buffered saline (PBS; pH 7.4) to a final concentration of approximately 1 × 10^6^ cells/mL. Cell lysis was performed by subjecting the samples to three consecutive freeze–thaw cycles, with freezing carried out at −80 °C. The resulting lysates were then centrifuged at 4000 rpm for 10 min at 4 °C, and the supernatants were collected and stored at −80 °C until subsequent analysis [35,36].

### 4.5. Biochemical Analysis

The intracellular protein levels of EGFR (Cat no: E0313Hu), MAPK (Cat no: e0873Hu), PI3K (Cat no: E0896Hu), NF-kB (Cat no: E0690Hu), caspase-7 (Cat no: E2257Hu), and caspase-3 (Cat no: E4804Hu) were determined in cell lysates using commercially available human ELISA kits (BTLab; Bioassay Technology Laboratory, Shanghai, China) in strict accordance with the manufacturer’s protocols. Briefly, 50 µL of the standards or samples were added to each well, followed by incubation with 10 µL of biotinylated detection antibody and 50 µL of horseradish peroxidase (HRP)-conjugated reagent. The plates were incubated at 37 °C for 1 h and washed five times to eliminate unbound material. Colorimetric signal development was achieved by the sequential addition of 50 µL each of substrate solutions A and B, with incubation at 37 °C for 10 min. The enzymatic reaction was terminated by adding 50 µL of stop solution, and absorbance was measured at 450 nm. Protein concentrations were calculated by reference to standard calibration curves [37].

### 4.6. Annexin V Binding Analysis

Apoptotic responses were quantified using the Muse^®^ Annexin V & Dead Cell Assay Kit (Luminex, Tokyo, Japan). Two experimental conditions were defined, comprising the untreated control cells and apigenin-treated cells. Following cellular adherence, cultures assigned to the treatment condition were exposed to apigenin-enriched culture medium for 24 h. Subsequent apoptosis analysis was performed in strict compliance with the manufacturer’s protocol, and quantitative data acquisition and analysis were carried out using the Guava^®^ Muse^®^ Cell Analyzer (Fremont, CA, USA) consistent with previously established methodologies [38].

### 4.7. Cell Cycle Analysis

Cell-cycle progression and arrest were analyzed using the Muse^®^ Cell Cycle Analysis Kit (Austin, TX, USA) in accordance with the manufacturer’s instructions. Both apigenin-treated and untreated control cells were collected by centrifugation at 300× *g* for 5 min and subsequently washed with ice-cold phosphate-buffered saline (PBS). Cells were gently vortexed and fixed in chilled 70% ethanol. Following fixation, samples were washed again with PBS and resuspended in Muse^®^ Cell Cycle Reagent (Part No. 4700-1495, Austin, TX, USA). Before acquisition, cell suspensions were incubated for 30 min at room temperature in the dark. The distribution of cells across the G_0_/G_1_, S, and G_2_/M phases was then quantified using the Muse^®^ Cell Analyzer (Millipore) (Fremont, CA, USA).

### 4.8. Immunofluorescence Staining Protocol

Cells were fixed in cold methanol at −20 °C for 20 min, followed by a PBS wash and blocking with 1% BSA for 1 h. Samples were then incubated overnight at 4 °C with a primary antibody cocktail of monoclonal EGFR (Santa Cruz, sc-373746) and polyclonal Caspase 3 (Thermo Fisher, PA5-114687, Waltham, MA, USA), both diluted 1/200.

After washing with PBS, the sections were incubated for 1 h at room temperature in the dark with secondary antibodies: Goat anti-Mouse Texas Red (Novus Bio, NBP1-73623, Centennial, CO, USA) and Goat Anti-Rabbit FITC (Jackson Immunoresearch, 111-095-003, West Grove, PA, USA), both diluted 1/100 in 1% BSA. Following a final rinse with distilled water, nuclei were counterstained with DAPI. Imaging was performed using a Zeiss Axiolab 5 fluorescence microscope equipped with an Axiocam 305 color camera and Colibri 3 light source. EGFR (red) and caspase-3 (green) immunoreactivity were quantified by measuring histogram values of fluorescence intensity across six random fields per sample using ImageJ software version 1.53k.

### 4.9. Statistical Analysis

Data are presented as the mean ± SEM of at least three independent experiments. Statistical analyses were performed using GraphPad Prism 10. Unpaired Student’s *t*-test was used for two-group comparisons, and one-way ANOVA with Tukey’s post hoc test was applied for three or more groups. IC_50_ values were calculated by nonlinear regression (log[inhibitor] vs. normalized response–variable slope). *p* < 0.05 was considered significant.

## 5. Conclusions

The present study demonstrates that apigenin exerts moderate antiproliferative and proapoptotic effects in hematologic malignancy cell models through coordinated modulation of the EGFR/MAPK and PI3K/Akt signaling pathways. Apigenin treatment reduced cell viability, induced apoptosis, and disrupted cell-cycle progression, accompanied by increased activation of caspase-3 and caspase-7 and by the suppression of key oncogenic signaling pathways, including EGFR, MAPK, PI3K, and NF-κB. The greater sensitivity observed in DOHH2 cells suggests that lymphoma cells may be more susceptible to inhibition of the apigenin-mediated pathway. These findings support the concept that apigenin acts as a multitarget anticancer compound that modulates multiple survival pathways simultaneously. The comparatively lower sensitivity of L929 normal cells further supports the potential selectivity of apigenin toward malignant cell populations. However, since the present results are based on in vitro experiments, further in vivo and translational studies are required to clarify the therapeutic potential of apigenin in hematologic malignancies.

## Data Availability

The original findings presented in this study are included in the article. For further inquiries, please contact the corresponding authors.

## Abbreviations

The following abbreviations are used in this manuscript:

CCK-8	Cell counting kit
CDKs	Cyclin-dependent kinases
CML	Chronic myeloid leukemia
DLBCL	Diffuse large B-cell lymphoma
DMSO	Dimethyl sulfoxide
EGFR	Epidermal growth factor receptor
EMT	Epithelial–mesenchymal transition
IC_50_	Half-maximal inhibitory concentration
MAPK	Mitogen-activated protein kinase
MMPs	Matrix metalloproteinases
NF-κB	Nuclear factor kappa B
PI3K	Phosphatidylinositol 3-kinase
R-CHOP	Rituximab-cyclophosphamide-hydroxydaunorubicin-oncovin-prednisone
PD-1/PD-L1	Programmed death-1/programmed death-ligand 1
TKIs	Tyrosine kinase inhibitors
